# Enzyme-Linked Immunosorbent Assay: An Adaptable Methodology to Study SARS-CoV-2 Humoral and Cellular Immune Responses

**DOI:** 10.3390/jcm11061503

**Published:** 2022-03-09

**Authors:** Amanda Izeli Portilho, Gabrielle Gimenes Lima, Elizabeth De Gaspari

**Affiliations:** 1 Immunology Center, Adolfo Lutz Institute, Sao Paulo 01246-902, SP, Brazil; a.izeliportilho@usp.br (A.I.P.); gabriellegimenes@usp.br (G.G.L.); 2 Graduate Program Interunits in Biotechnology, University of Sao Paulo, Sao Paulo 05508-900, SP, Brazil

**Keywords:** ELISA, immune response, antibody, cytokine, SARS-CoV-2

## Abstract

The Enzyme-Linked Immunosorbent Assay is a versatile technique, which can be used for several applications. It has enormously contributed to the study of infectious diseases. This review highlights how this methodology supported the science conducted in COVID-19 pandemics, allowing scientists to better understand the immune response against SARS-CoV-2. ELISA can be modified to assess the functionality of antibodies, as avidity and neutralization, respectively by the standardization of avidity-ELISA and surrogate-neutralization methods. Cellular immunity can also be studied using this assay. Products secreted by cells, like proteins and cytokines, can be studied by ELISA or its derivative Enzyme-linked immunospot (ELISpot) assay. ELISA and ELISA-based methods aided the area of immunology against infectious diseases and is still relevant, for example, as a promising approach to study the differences between natural and vaccine-induced immune responses against SARS-CoV-2.

## 1. Introduction

Enzyme-Linked Immunosorbent Assay (ELISA) is a widely used method, most of all, because of its flexibility: in-house assays can be performed or commercial kits can be used; it is possible to analyze several samples because of its high throughput capacity and lots of different analytes can be studied. Therefore, as long as it is standardized, the test has multiple applications [1].

When it comes to the immune response in the context of infectious diseases, ELISA is a technique that can provide a variety of data. For example, it supports diagnosis detecting microbial antigens in a sample; verifies prior exposure to the pathogen detecting antibodies; underpins epidemiologic monitoring with serologic surveys; and aids immune intervention studies, through lots of applications (e.g., supporting vaccine studies or filtering monoclonal antibodies) [2,3,4].

The aim of this review is to discuss how ELISA and ELISA-based technology were used to respond to COVID-19 pandemics. Also, it will describe some features of the humoral and cellular response against SARS-CoV-2 that were elucidated using such techniques.

## 2. Detection of Immune Response to SARS-CoV-2

Several tests can be used to investigate the immune response. Choosing the more adequate assay should consider laboratory facilities, regarding both infrastructure and professional qualifications, as well as the question to be answered. Understanding the advantages and limitations of a serologic technique is the key not just to making the right choice, but also to address an adequate analysis [5,6,7].

## 3. Enzyme-Linked Immunosorbent and Other Immunoassays

Several immunoassays are used to study the immune response after vaccination or natural infection. In the case of ELISA, it allows high throughput flows, supported by automatic washers and readers. Usually, it is performed in 96-wells microplates. The first manuscripts proposing its use in SARS-CoV-2 studies consisted of protocols standardized in house [8,9]. The interesting point of such protocols is the possibility of re-standardizing it in different laboratories. However, commercial kits are also available, addressing the needs of clinical laboratories [10].

Chemiluminescence immunoassays allow complete automation and several kits are available now. However, a study found good correlation between ELISAs protocols and automated immunoassays, using both Nucleocapsid and Spike proteins as coating antigens [11].

ELISA can also be applied for multiplex analysis, using microarrays where different antigens are coated. Two examples are ViraChip^®^ (ViraMed Biotech AG, Planegg, Niemcy) and multiSero assays, which use, respectively, subunits 1 (S1) and 2 (S2) of Spike (S) and Nucleocapsid (N) and S, N and Receptor-binding domain (RBD) of SARS-CoV-2. Reports of these tests reach 95 (multiSero) and 100% specificity (ViraChip^®^, ViraMed Biotech AG, Planegg, Niemcy) [12,13,14,15]. Thus, the results obtained distinguish the antigens triggering the humoral response, which supports immunologic and vaccine studies.

Usually, ELISA or chemiluminescence assays are more sensitive than immunocromatography [15,16]. However, a study found that the sensitivity of lateral flow immunoassays ranged from ≥92.1 to 100% when samples collected 14 days after the infection were tested [17]. It must be highlighted that sensitivity of immunoassays is related to sampling time. It might decrease when samples collected after several months are used, which seems to be a feature of SARS-CoV-2 immune response [18].

All in all, ELISA, as well as other techniques adapted from it, are capable of detecting the presence of SARS-CoV-2 antigens or antibodies in the sample tested. All these platforms are easy to perform and can provide results within hours. Hence, they can be used for diagnostics or research purposes [1,19]. Also, the methodologies can be adapted to describe other features of the immune response, as cytokine release, which helps to elucidate SARS-CoV-2 immune response. It is important to note that the question to be answered should guide both choice and standardization of the assay [2,5].

## 4. SARS-CoV-2 Antigens and Antibodies

The main antigens studied in SARS-CoV-2 humoral response are structural proteins Spike (S) and Nucleocapsid (N). It is observed that S protein can be detected entirely, or by means of its subunits 1 (S1) and 2 (S2). Another antigen related to it is the Receptor-binding domain (RBD), which lies at S1 [20].

Before discussing the antigens and immunoglobulin (Ig) classes, it is relevant to highlight that, in general, the detection of antibodies is a limited tool for diagnosis. In some cases, the detection of IgM or IgG may point out acute or chronic infection, respectively [21]. However, the pathophysiology of the disease should be well-known, so the results will not be masked by vaccine response or immunologic window. This is why detecting the pathogen itself, its nucleic acid, or antigens usually are considered a gold standard [22,23]. Concerning SARS-CoV-2, the gold standard for diagnosis is the detection of viral RNA by RTq-PCR. Considering the unprecedented number of cases, the use of antigen-detection was a strategy to support COVID-19 diagnosis, proving the presence of viral antigens in nasopharyngeal samples [23,24].

The abundance of S and N antigens in the course of SARS-CoV-2 infection substantiates its use in immunoassays, regardless of the aim being to detect the antigen or the antibodies [25]. It must be noted that there is a difference in the positivity dynamics of each antigen. Anti-N antibodies become positive, on average, two days earlier than anti-S antibodies, but they have a shorter half-life. In fact, anti-S antibodies are detectable for longer periods. Thus, it might explain decreased sensitivity of N-based assays when samples collected at longer moments are tested [26].

Antigen-detection assays are often based on lateral-flow immunoassays using nasopharyngeal samples, where the viral load is concentrated. The use of two antigens increases test sensitivity [23]. In fact, testing nasopharyngeal samples in an N-chemiluminescence enzymatic immunoassay provided satisfactory concordance with gold-standard RT-qPCR [27]. To achieve high sensitivity and specificity, antigen-detection tests should be conducted up to seven days after infection [28]. However, a fluorescent ELISA to detect the Nucleocapsid antigen in serum was developed, as well as an ELISA to capture the Spike protein [7,29]. To improve clinical-laboratory testing, automated assays to detect SARS-CoV-2 antigens are available as well, and correlated well with RT-qPCR results. In that case, specificity may reach 100%, while sensitivity levels are related to the positivity threshold established [30].

Considering antibody-detection assays, it should be understood that using S and N proteins might increase the sensitivity, providing more epitopes for the antibodies to bind. On the other hand, it can increase the cross-reactivity. Thus, subunit antigens, such as RBD, have been used to assess SARS-CoV-2 immune response in a more specific way [25]. This antigen is undeniably important, since it mediates the interaction between the virus and the Angiotensin-converting enzyme 2 (ACE-2), used as receptor. However, there are studies reporting neutralizing antibodies directed to the N-terminal domain (NTD) of S1 subunit, highlighting that this is another relevant antigen to be studied [31,32].

Envelope (E) and Membrane (M) are other structural proteins but, due to their small molecular size, usually they do not provide a robust humoral response [20]. However, recent studies provide new data about it. Anti-Membrane antibodies were detected in COVID-19 patients up to 12 months after infection and Envelope-IgM has shown a less accentuated decrease than S1-IgM [33]. Thus, tests detecting Ig against different antigens could be employed to distinguish natural and vaccine-induced immune responses, as long as the subjects were vaccinated with subunit vaccines.

Considering Ig classes, the curve of anti-SARS-CoV-2 antibodies differs from most infections, making it difficult to predict acute or chronic/memory immune responses. Usually, IgM antibodies increase during the first two weeks of infection; hence, its “acute marker” characteristic was coined. Afterwards, IgM antibodies decay while IgG titers increase [21]. In COVID-19, the first reports suggested the same pattern, but more recent studies point to a different one, where IgM and IgG antibodies increase concomitantly since the beginning of infection, being detected as early as three days following infection [34,35]. Even more curious is the fact that IgA would be a better marker of acute infection than IgM, increasing in the first days following infection and maintaining its titers for up to 30 days, whereas IgM would decay after 15 days. Also, combined detection of IgA/IgG would confer more sensitive results than IgM/IgG. Another way to increase the sensitivity of immunoassays is to use anti-Ig detection antibodies, which will reveal the presence of the whole antibody content of the sample, regardless of the Ig class [36].

IgG is related to immunologic memory and, in some cases, it can be detected years after the infection or vaccination [21]. However, serologic studies suggest that anti-SARS-CoV-2 IgG would decrease six to eight months after infection [37,38]. Although the vaccination campaigns are recent and studies to elucidate the immunologic memory conferred by them are still ongoing, there are reports showing persistence of antibodies up to six months after vaccination [39,40]. It is worth emphasizing that seropositivity for anti-SARS-CoV-2 IgM and IgG occurs about the same time following infection.

**Figure 1:**
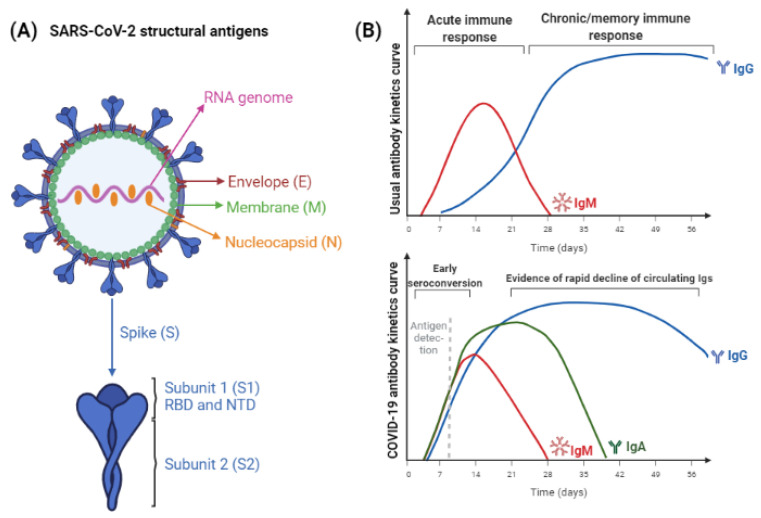
(**A**) SARS-CoV-2 structural antigens, used for immunoassays. Nucleocapsid and Spike proteins are the most abundant antigens, therefore, they are used for improved sensitivity. Antigen-subunits, as S1 and S2, or RBD and NTD, can provide more specific results. (**B**) The expected IgM/IgG curves following exposure to antigen through natural infection or vaccination. The maturation of immune response usually leads to initial IgM induction and, while titers of this antibody class decrease, IgG titers increase and persist for longer periods, which varies according to the pathogen considered. Below it, kinetics of SARS-CoV-2 antigens and antibodies detection is represented. Differently from the expected, titers of IgA, IgM, and IgG antibodies increase in parallel. The time to achieve seronegativity and the particularities of vaccine-induced humoral response are still being elucidated (figure created with BioRender).

Figure 1 represents the structural antigens used in SARS-CoV-2 immunoassays and the antigen or antibody detection curve expected in SARS-CoV-2 immune response.

The sole presence of antibodies following natural infection should be cautiously analyzed, especially when the patient is individually considered, because it does not prove an antibody functionality, predicting protection [2]. So far, high antibodies quantities were observed in severe COVID-19 cases [41,42].

For some infectious diseases, the specificity of the immune response can predict better or worse prognostic. Some studies following COVID-19 patients suggested a worst prognosis when a Nucleocapsid was observed instead of a Spike-biased immune response. However, others could not observe any difference between the antigens recognized and the severity of the disease [41,42]. Similarly, the impact of the kinetics of humoral response was related to a better or worst clinical condition. Hashem et al. [43] reported that an early seroconversion correlated with fatality, but Lucas et al. [44] observed a delayed antibody response in deceased patients. Meanwhile, the study of Van Elslande et al. [17] did not indicate significant differences in time to critical and non-critical patients to seroconvert. Disagreeing results suggest that further studies are necessary to elucidate these aspects and support the interpretation of ELISA results.

Secretory IgA, locally produced at mucosal sites, are chief to control mucosal infections, but the role of seric IgA has been up for debate. It may help control the inflammation, but authors disagree about its capacity to activate the complement system and opsonize pathogens [45,46,47]. Regarding COVID-19, there is evidence that seric IgA acts on early virus neutralization [48]. Fedele et al. [49] observed that early IgA response was seen in patients with less severe COVID-19, but Portilho et al. [42] could not relate increased IgA response to mild patients response. Despite the disagreeing results of different studies, the detection of seric IgA could reflect secretory IgA. There is evidence that naïve B cells activated in the mucosa can be home to the marginal zone of the spleen, so IgA triggered against mucosal pathogens could be released in the bloodstream and contribute to control the infection [50,51].

Finally, even though antibody-detection assays are limited to diagnosing the current infection, these technologies are still relevant, especially to select plasma donors, vaccine studies, and epidemiological monitoring [2].

## 5. Functionality of Antibodies

**Figure 2:**
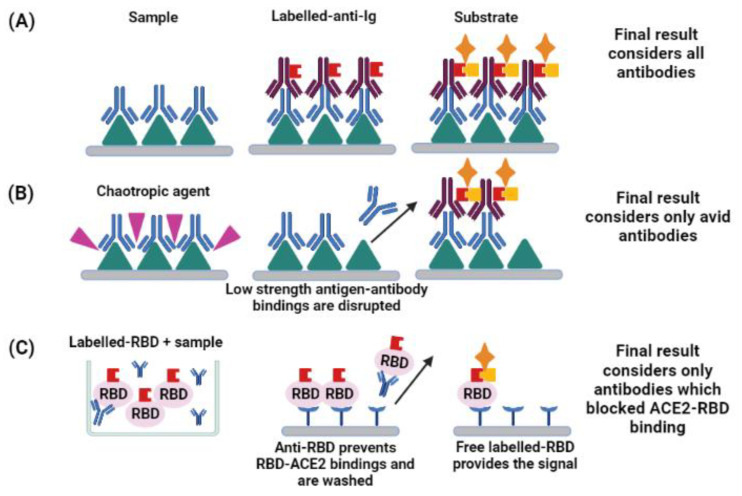
Differences in non-functional and functional enzyme-linked assays. While non-functional assays (**A**), like regular ELISA, detect antibodies that are bound with the antigen, functional assays detect antibodies regarding specific characteristics. (**B**) Avidity assays consist in adding a chaotropic agent that will disturb the epitope-paratope interaction, disrupting weak bindings. Only avid antibodies keep attached and are revealed in the final result. Both A and B assays should consider which antigen of SARS-CoV-2 was used to coat the plate (e.g., RBD, Spike protein, Nucleocapsid protein) and the specificity of the anti-Ig used for detection (e.g., anti-IgA, anti-IgM, anti-IgG). For quantitative results, the higher the signal, the higher the antibody quantity. (**C**) Surrogate-virus neutralization assays, based on a competitive ELISA, allow the interaction between labelled-RBD and the sample, after that, the mix is incubated in ACE-2 coated plates. Therefore, free-RBD binds to the plate and complexes antibody-labelled-RBD, which cannot bind with it, are washed. It should be noted that the assay considers all anti-RBD antibodies, despite its Ig class. For quantitative results, the lower the signal, the higher is the antibody quantity. (figure created with BioRender).

Despite the technical differences of each type of ELISA described so far, (as coating-antigen, class of immunoglobulin detected, the sample used and overall sensibility and specificity), all of them would suggest the sole presence of antibodies [2,5]. Positive results in such assays should be carefully interpreted, since it indicates past exposure to the virus rather than protection [2]. To describe the functionality of antibodies, other ELISA-derived assays are more suitable, like avidity and neutralization, as represented in Figure 2.

The affinity maturation is a well-known feature of the immune response. Once the antigen is presented to B lymphocytes, Immunoglobulin (Ig) gene segments undergo somatic mutation. It leads to Ig-class switch and to the differentiation of B lymphocytes in plasma cells or memory cells. These rearrangements happen in the germinal centers and aim to select the higher-quality antibodies for immune response [52]. A characteristic of the improved quality of these Igs is increased affinity and avidity. The first relates to the interaction between monovalent epitope and paratope, whereas the latter results from the multivalent interactions between antigen and antibody [53].

Therefore, avidity assays distinguish binding strength of antibodies to the antigen. In the case of SARS-CoV-2 infection, if its avidity index is high, the antibody might be more effective to block the virus–receptor interaction, thus, being able to control the viral infection. On the other hand, low-avidity is one of the causes of antibody-dependent enhancement, which favors infection and increases inflammation [54,55]. Assessing the avidity of anti-SARS-CoV-2 antibodies provides more robust data, so the maturation of humoral response and antibody functionality in COVID-19 immune response can be described [54]. A modified-ELISA can be used to assess the avidity index of antibodies. It consists of adding a chaotropic agent, such as urea or potassium thiocyanate, along with serum incubation or right after it. The chaotropic agents are able to disrupt hydrophobic interaction, hydrogen bonds, van der Waals forces, or even electrostatic interactions, which will impair the antigen-antibody interaction. As a result, only antibodies with increased avidity will remain bounded to the antigen [53].

Neutralizing antibodies (Nabs) can inhibit the infection, acting by different pathways: directing degradation of the pathogen; inducing viral aggregation; compromising key processes, like replication and transcription; or blocking the virus-receptor binding [56]. Given that SARS-CoV-2 internalization is mediated by the interaction between RBD and ACE2, antibodies that inhibit this binding are potentially neutralizing [20]. Therefore, surrogate-virus neutralization tests (sVNT) check if serum sample can inhibit RBD-ACE2 interaction and were standardized using a competitive ELISA approach [57]. Interestingly, this assay allows to test samples for neutralizing antibodies without special requirements of biosafety level, cost and training, thus being suitable for automatization [58,59].

As described before, even though the main mechanisms of neutralizing antibodies are blocking the virus-receptor binding, others are known [56]. To assess the neutralization capacity of a sample, regardless of the mechanism of action of the antibodies, Ig class and epitope specificity, the conventional live-virus neutralization test (cVNT) should be applied. It consists of mixing antibody sample and live virus, then incubating it with a susceptible host (chicken-eggs or cell lines, for example), allowing to observe cytopathic effects or plaque formation, indicatives of neutralizing activity [57]. Despite the in-depth and accurate results, cVNT is more expensive, require well-trained professionals and must be proceeded in biosafety level 3 laboratories, which limits its use [57]. There are reports of correlated results obtained by cVNT and sVNT [58,59], but other studies suggested an overestimation or an underestimation of neutralizing titers by sVNT [60,61,62]. Such disagreements indicate that sVNT results should be analyzed considering the particularities of the scenario where it was conducted, but thi methodology is a relevant option to expand neutralizing data about SARS-CoV-2 immune response.

## 6. Cellular Immunity

**Figure 3:**
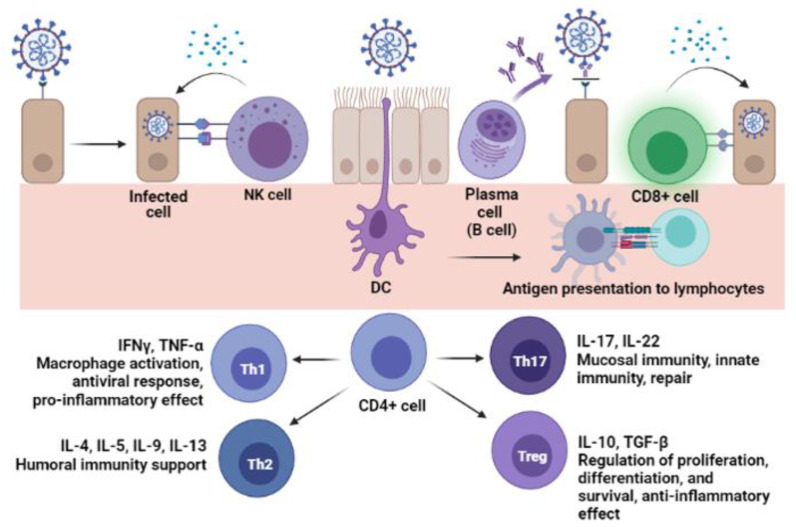
Immune cells and SARS-CoV-2 infection. SARS-CoV-2 is internalized after the RBD-ACE2 interaction. Natural-killer (NK) cells can identify infected cells and kill them after the release of cytotoxic proteins (like perforin and granzyme). Dendritic cells (DC) are responsible for capture, processing, and presentation of pathogen to lymphocytes, to assemble the adaptive immune response. After activation and antigen presentation, plasma (B) cells produce and release antibodies that, for example, can neutralize the virus by inhibition of RBD-ACE2 interaction; while cytotoxic T cells (CD8+ cell) recognize infected cells and kill it by releasing cytotoxic proteins. Auxiliary T cells (CD4+ cell) differentiate to certain T helper (Th) types, releasing cytokines that modulate the immune environment, supporting different arms of response. A well-orchestrated immune response is necessary to control the infection without harming the host and the products secreted by cells (e.g., proteins, cytokines, immunoglobulins) can be enzyme-linked detected. (figure created with BioRender).

Cellular immunity is key to understand the immune response: innate cells can directly neutralize pathogens; also, they perform antigen capture and presentation. Adaptive cells can kill the pathogen and infected cells and support antibody production. Thus, the release of cytokines orchestrates the overall immune response [21,63]. Figure 3 shows the contribution of immune cells in SARS-CoV-2 infection.

Although cellular immunity is needed to control the infection, its assessment through assays like flow cytometry is more expensive, require trained professionals to proceed with data analysis, and it is less prone to automatization [6]. Hence, these tests are less suitable for routine or studies of low-income settings [5,64]. To overcome this barrier, the cytokine secreted by cells or present in serum samples can be enzyme-linked detected, for instance, using ELISA plates coated with capture antibodies. It is possible to predict the cellular population acting on the immune response according to its secretory profile [64,65].

The Enzyme-Linked Immunosorbent Assay, on the other hand, is an adaptation of ELISA firstly proposed by Czerkinsky and colleagues, which can be used to study cellular response [66,67]. Briefly, it consists of cultivating a cell suspension in a microplate covered by a membrane coated with capture antibodies, where the antibodies or cytokines secreted by the cells will bind. After that, the reaction is revealed using precipitating substrates [68]. It is more expensive than regular ELISA, especially when commercial kits are used. Thus, it is less prone to automatization and requires training to work with cellular culture. However, it can also be adapted to different infrastructures and the technique is highly sensitive, being used especially for vaccine investigations [68,69].

Differently from phenotyping by Flow cytometry or Immunohistochemistry, cytokine-ELISA and ELISpot results will render the functionality of the cell population, showing which are the products secreted by the cells [68].

In that way, we must understand the cytokine role to interpret cytokine-ELISA results. Once it is known, the cellular population responsible for the cytokine release can be predicted, as well as the effects it will render. Table 1 summarizes the features of the main cytokines studied in SARS-CoV-2 response and its effects. It is worth mentioning that the role of some cytokines, such as IL-17, is yet to be elucidated, since disagreeing results have been reported.

The studies of cytokine secretion contributed to the clinical management of COVID-19 cases. Cytokine storm, marked by increased levels of IL-1β, IL-6, and TNF-α, is an issue for COVID-19 patients, leading to immune hyperactivation, thus harming the host. Once it was verified, therapy with monoclonal antibodies started to be investigated, aiming for the reduction of cytokine activity and, consequently, reduction of inflammation [76].

It is described that viral immune response leads to a co-effort between antibodies and cytotoxic cells to limit viral spread and clear the infection [77,78]. So, ideally, a SARS-CoV-2 vaccine should activate B and T lymphocytes. Also, the differentiation of CD4+ cells into Th1 type would be desirable to increase IFN-Υ secretion, since this cytokine improves intracellular killing of pathogens [79]. Thus, there is evidence that convalescent patients with undetectable antibody titers were able to control COVID-19 due to cellular immunity [80].

Therefore, it would be important to study cytokine secretion in SARS-CoV-2 vaccine development. Pfizer and AstraZeneca/Oxford vaccines trials checked IFN-Υ secretion, while Janssen studied both IFN-Υ and IL-4 secretion by ELISpot. Results supported that these vaccines could guarantee the ideal cytokine secretion to prevent COVID-19 [81,82,83]. Simons et al. [84] observed that B-cell depleted patients could control SARS-CoV-2, possibly due to T cell response, which was assessed through ELISpot analysis.

## 7. Enzyme-Linked Techniques as Tools to Study Natural Infection and Vaccination

There is an increase in studies comparing the response elicited by natural SARS-CoV-2 infection or vaccination, which is key to understand the protective response and refine the novel generation of COVID-19 vaccines. ELISA and its derivate techniques can aid such studies.

As emphasized previously, even though cellular assays could provide more robust data, antibody measurement is easy to perform and useful for clinics [85].

Avid antibodies are known to be more effective in controlling viral infections, resulting from adequate affinity maturation. It was proposed that, similarly to other coronaviruses, the decay of antibodies and failure to achieve high avidity in response to SARS-CoV-2 infection would guarantee immune evasion for the virus, thus allowing reinfections [86]. There are studies pointing to low and intermediate avidity indexes following SARS-CoV-2 infection that support this assumption [9,87]. On the other hand, studies suggested an affinity maturation of antibodies after two doses of COVID-19 vaccines [59]. Two doses of mRNA and vector vaccines induced high avidity IgG [88]. The facility to freeze serum samples compared with immune cells, along with the resistance of antibodies to freeze-thaw processes, is another issue to be considered, facilitating the comparisons with serum samples collected before COVID-19 vaccination campaigns [89].

## 8. Conclusions

Enzyme-linked assays have supported several scientific studies so far. Currently, these methodologies had been exhaustively used to investigate the immune response to SARS-CoV-2. However, the type of ELISA or ELISA-based applied must consider the question to be answered. Addressing the questions with clarity is important to compare results and improve the scientific discussion. The differences between the immune response of natural infection and vaccination are a promising field for study—and might be aided by this adaptable, versatile technique.

## Figures and Tables

**Table 1:** Cytokines studied in SARS-CoV-2 immune response, the cell responsible for its secretion, immune function of the cytokine, effects modulated in the host and relations established in COVID-19 response.

Cytokine	Secretor Cell	Immune Function	Effect on the Host	SARS-CoV-2 Association
CXCL-10	Monocytes	Monocytes, macrophages, NK cells, DCs and LT chemotaxis	Inflammation	Severe disease [70]
Interferon-gamma (IFN-Υ)	NK cells and LT-CD4+ (Th1)	IL-4 inhibition, Th1 differentiation, increased MHC I and II expression	Inflammation and anti-viral immune response	Lung injury [71]
IL-1β	Macrophages	LB proliferation and differentiation, phagocytes stimulation	Inflammation	Decreased oxygen saturation, poor outcome [72,73]
IL-2	Activated LT-CD4+, LB and monocytes	NK and T cell activation and proliferation, B cell activation along with IL-4	Inflammation and antigen-specific stimulation	ICU-hospitalization [35,72]
IL-4	LT-CD4+ (Th2)	LB differentiation and proliferation, increased expression of MHC-II	Antigen-specific humoral response	Mild disease [70]
IL-6	Lymphocytes and monocytes	Increased acute inflammation-cytokines release, eosinophil chemotaxis	Immune modulation (pro or anti-inflammatory), antigen-specific response, and anti-viral response	Decreased oxygen saturation, poor outcome, increased risk of death [73,74]
IL-8	Macrophages	Neutrophil and granulocytes chemotaxis, phagocytosis stimulation	Inflammation	Severe disease, increased risk of death [74,75]
IL-10	LT-CD8+	Inhibition of Th1 cytokines, decreased cytolytic response	Inflammation	Severe disease and ICU hospitalization [35,75]
IL-17	LT-CD4+ (Th17)	Neutrophil activation	Inflammation, mucosal activation, tissue repair	Decreased oxygen-saturation and lung injury [71,73], mild disease [70]
Tumor-necrosis factor (TNF)-α	Macrophages	Phagocytes chemotaxis and phagocytosis stimulation	Inflammation	Severe disease, ICU hospitalization, and increased risk of death [35,74,75]

## Data Availability

No new data were created or analyzed in this study. Data sharing is not applicable to this article.

## References

[1] Crowther J.R. (2009). The ELISA Guidebook.

[2] West R., Kobokovich A., Connell N., Gronvall G.K. (2021). COVID-19 Antibody Tests: A Valuable Public Health Tool with Limited Relevance to Individuals. Trends Microbiol..

[3] Maggi E., Canonica G.W., Moretta L. (2020). COVID-19: Unanswered questions on immune response and pathogenesis. J. Allergy Clin. Immunol..

[4] Assadiasl S., Fatahi Y., Zavvar M., Nicknam M.H. (2020). COVID-19: Significance of antibodies. Hum. Antibodies.

[5] Ong D.S.Y., Fragkou P.C., Schweitzer V.A., Chemaly R.F., Moschopoulos C.D., Skevaki C., European Society of Clinical Microbiology and Infectious Diseases (ESCMID) Study Group for Respiratory Viruses (ESGREV) (2021). How to interpret and use COVID-19 serology and immunology tests. Clin. Microbiol. Infect..

[6] Hamilton R.G. (1994). The clinical immunology laboratory of the future. Clin. Chem..

[7] Liu G., Rusling J.F. (2021). COVID-19 Antibody Tests and Their Limitations. ACS Sensors.

[8] Amanat F., Stadlbauer D., Strohmeier S., Nguyen T.H.O., Chromikova V., McMahon M., Jiang K., Arunkumar G.A., Jurczyszak D., Polanco J. (2020). A serological assay to detect SARS-CoV-2 seroconversion in humans. Nat. Med..

[9] Moura A.D., Costa H.H.M., Correa V.A., Lima A.K.S., Lindoso J.A.L., De Gaspari E., Hong M.A., Cunha-Junior J.P., Prudencio C.R. (2021). Assessment of avidity related to IgG subclasses in SARS-CoV-2 Brazilian infected patients. Sci. Rep..

[10] Allinson J.L. (2011). Automated immunoassay equipment platforms for analytical support of pharmaceutical and biopharmaceutical development. Bioanalysis.

[11] Van Elslande J., Decru B., Jonckheere S., Van Wijngaerden E., Houben E., Vandecandelaere P., Indevuyst C., Depypere M., Desmet S., André E. (2020). Antibody response against SARS-CoV-2 spike protein and nucleoprotein evaluated by four automated immunoassays and three ELISAs. Clin. Microbiol. Infect..

[12] Byrum J.R., Waltari E., Janson O., Guo S.-M., Folkesson J., Chhun B.B., Vinden J., Ivanov I.E., Forst M.L., Li H. (2021). multiSero: Open multiplex-ELISA platform for analyzing antibody responses to SARS-CoV-2 infection. MedRxiv.

[13] Krüttgen A., Cornelissen C.G., Dreher M., Hornef M., Imöl M., Kleines M. (2020). Comparison of four new commercial serologic assays for determination of SARS-CoV-2 IgG. J. Clin. Virol..

[14] Olbrich L., Castelletti N., Schälte Y., Garí M., Pütz P., Bakuli A., Pritsch M., Kroidl I., Saathoff E., Guggenbuehl Noller J.M. (2021). Head-to-head evaluation of seven different seroassays including direct viral neutralisation in a representative cohort for SARS-CoV-2. J. Gen. Virol..

[15] Bastos L.M., Tavaziva G., Abidi S.K., Campbell J.R., Haraoui L.P., Johnston J.C., Lan Z., Law S., MacLean E., Trajman A. (2020). Diagnostic accuracy of serological tests for covid-19: Systematic review and meta-analysis. BMJ.

[16] Saker K., Escuret V., Pitiot V., Massardier-Pilonchéry M., Paul S., Mokdad B., Langlois-Jaques C., Rabilloud M., Goncalves D., Fabien N. (2021). Evaluation of commercial anti-SARS-CoV-2 antibody assays and comparison of standardized titers in vaccinated health care workers. J. Clin. Microbiol..

[17] Van Elslande J., Houben E., Depypere M., Brackenier A., Desmet S., André E., van Ranst M., Lagrou K., Vermeersch P. (2020). Diagnostic performance of seven rapid IgG/IgM antibody tests and the Euroimmun IgA/IgG ELISA in COVID-19 patients. Clin. Microbiol. Infect..

[18] Perez-Saez J., Zaballa M.E., Yerly S., Andrey D.O., Meyer B., Eckerlle I., Balavoine J.-F., Chappuis F., Pittet D., Trono D. (2021). Persistence of anti-SARS-CoV-2 antibodies: Immunoassay heterogeneity and implications for serosurveillance. Clin. Microbiol. Infect..

[19] Dowlatshahi S., Shabani E., Abdekhodaie M.J. (2021). Serological assays and host antibody detection in coronavirus-related disease diagnosis. Arch. Virol..

[20] Dai L., Gao G.F. (2021). Viral targets for vaccines against COVID-19. Nat. Rev. Immunol..

[21] Mesquita Júnior D., Araújo J.A.P., Catelan T.T.T., Souza A.W.S., Cruvinel W.M., Andrade L.E.C., Silva N.P. (2010). Immune System Part II—Basis of the immunological response mediated by T and B lymphocytes. Brazilian J. Rheumatol..

[22] Cancrini G., Iori A. (2004). Traditional and innovative diagnostic tools: When and why should be applied. Parassitologia.

[23] Ilkhani H., Hedayat N., Farhad S. (2021). Novel approaches for rapid detection of COVID-19 during the pandemic: A review. Anal. Biochem..

[24] Matsuda E.M., de Campos I.B., de Oliveira I.P., Colpas D.R., dos Santos Carmo A.M., Brígido L.F.M. (2021). Field evaluation of COVID-19 antigen tests versus RNA based detection: Potential lower sensitivity compensated by immediate results, technical simplicity, and low cost. J. Med. Virol..

[25] Khan J., Al Asoom L.I., Khan M., Chakrabartty I., Dandoti S., Rudrapal M., Zothantluanga J.H. (2021). Evolution of RNA viruses from SARS to SARS-CoV-2 and diagnostic techniques for COVID-19: A review. J. Basic Appl. Sci..

[26] Lumley S.F., Wei J., O’Donnel D., Stoesser N.E., Matthews P.C., Howarth A., Hatch S.B., Marsden B.D., Cox S., James T. (2021). The Duration, Dynamics, and Determinants of Severe Acute Respiratory Syndrome Coronavirus 2 (SARS-CoV-2) Antibody Responses in Individual Healthcare Workers. Clin. Infect. Dis..

[27] Villalta D., Moratto A., Salgarolo V., Da Re M., Giacomello R., Malipiero G. (2022). New-Generation Quantitative Immunoassays for SARS-CoV-2 Antibody Detection: Need for Harmonization. Ann. Lab. Med..

[28] Abusrewill Z., Alhudiri I.M., Kaal H.H., El Meshri S.E., Ebrahim F.O., Dalyoum T., Efrefer A.A., Ibrahim K., Elfghi M.B., Abusrewill S. (2021). Time scale performance of rapid antigen testing for SARS-CoV-2: Evaluation of 10 rapid antigen assays. J. Med. Virol..

[29] Girt G.C., Lakshminarayanan A., Huo J., Dormon J., Norman C., Afrough B., Harding A., James W., Owens R.J., Naismith J.H. (2021). The use of nanobodies in a sensitive ELISA test for SARS-CoV-2 Spike 1 protein. R. Soc. Open Sci..

[30] Van der Moeren N., Zwart V.F., Goderski G., Rijkers G.T., van den Bijllaardt W., Veenemans J., Kluytmans J.A.J.W., Pas S.D., Meijer A., Verweij J.J. (2021). Performance of the Diasorin SARS-CoV-2 antigen detection assay on the LIAISON XL. J. Clin. Virol..

[31] Amanat F., Thapa M., Lei T., Ahmed S.M.S., Adelsberg D.C., Carreño J.M., Strohmeier S., Schmitz A.J., Zafar S., Zhou J.Q. (2021). SARS-CoV-2 mRNA vaccination induces functionally diverse antibodies to NTD, RBD, and S2. Cell.

[32] Krut V.G., Astrakhantseva I.V., Chuvpilo S.A., Efimov G.A., Ambaryan S.G., Drutskaya M.S., Nedospasov S.A. (2021). Antibodies to the N-Terminal Domain of Angiotensin-Converting Enzyme (ACE2) That Block Its Interaction with SARS-CoV-2 S Protein. Dokl. Biochem. Biophys..

[33] Amjadi M.F., Adyniec R.R., Gupta S., Bashar S.J., Mergaert A.M., Braun K.M., Moreno G.K., O’Connor D.H., Friedrich T.C., Safdar N. (2021). Anti-membrane and anti-spike antibodies are long-lasting and together discriminate between past COVID-19 infection and vaccination. medRxiv.

[34] Zhao J., Yuan Q., Wang H., Liu W., Liao X., Su Y., Wang X., Yuan J., Li T., Li J. (2020). Antibody Responses to SARS-CoV-2 in Patients with Novel Coronavirus Disease 2019. Clin. Infect. Dis..

[35] Huang C., Wang Y., Li X., Ren L., Zhao J., Hu Y., Zhang L., Fan G., Xu J., Gu X. (2020). Clinical features of patients infected with 2019 novel coronavirus in Wuhan, China. Lancet.

[36] Ma H., Zeng W., He H., Zhao D., Jiang D., Zhou P., Cheng L., Li Y., Ma X., Jin T. (2020). Serum IgA, IgM, and IgG responses in COVID-19. Cell. Mol. Immunol..

[37] Liu C., Yu X., Gao C., Zhang L., Zhai H., Hu Y., Liu E., Wang Q., Gao Y., Wei D. (2021). Characterization of antibody responses to SARS-CoV-2 in convalescent COVID-19 patients. J. Med. Virol..

[38] Dan J.M., Mateus J., Kato Y., Hastie K.M., Yu E.D., Faliti C.E., Grifoni A., Ramirez S.I., Haupt S., Frazier A. (2021). Immunological memory to SARS-CoV-2 assessed for up to 8 months after infection. Science.

[39] Swadzba J., Anyszek T., Panek A., Martin E. (2021). Anti-Spike SARS-CoV-2 IgG Assessment with a Commercial Assay during a 4-Month Course after COVID-19 Vaccination. Vaccines.

[40] Ciabattini A., Pastore G., Fiorino F., Polvere J., Lucchesi S., Pettini E., Stefano A., Rancan I., Durante M., Miscia M. (2021). Evidence of SARS-CoV-2-Specific Memory B Cells Six Months After Vaccination With the BNT162b2 mRNA Vaccine. Front. Immunol..

[41] Atyeo C., Fischinger S., Zohar T., Slein M.D., Burke J., Loos C., McCulloch D.J., Newman K.L., Wolf C., Yu J. (2020). Distinct Early Serological Signatures Track with SARS-CoV-2 Survival. Immunity.

[42] Portilho A.I., Silva V.O., Ahagon C.M., Matsuda E.M., de Oliveira E.L., da Silveira E.P.R., Lima A.K.S., Lindoso J.A.L., Campos I.B., Hong M.A. (2021). Humoral response to spike S1 and S2 and nucleocapsid proteins on microarray after SARS-CoV-2 infection. J. Med. Virol..

[43] Hashem A., Algaissi A., Almahboub S., Alfaleh M., Abujamel T., Alamri S., Alluhaybi K., Hobani H., AlHarbi R., Alsulaiman R. (2020). Early Humoral Response Correlates with Disease Severity and Outcomes in COVID-19 Patients. Viruses.

[44] Silva J., Lucas C., Sundaram M., Israelow B., Wong P., Klein J., Lu P., Venkataraman A., Liu F., Mao T. (2021). Saliva viral load is a dynamic unifying correlate of COVID-19 severity and mortality. medRxiv.

[45] Mestecky J., Mcghee J.R. (1987). Immunoglobulin A (IgA): Molecular and Cellular Interactions Involved in IgA Biosynthesis and Immune Response. Adv. Immunol..

[46] Kerr M.A. (1990). The structure and function of human IgA. Biochem. J..

[47] Hiemstra P.S., Gorter A., Stuurman M.E., van Es L.A., Daha M.R. (1987). Activation of the alternative pathway of complement by human serum IgA. Adv. Exp. Med. Biol..

[48] Sterlin D., Mathian A., Miyara M., Mohr A., Anna F., Claër L., Quentric P., Fadlallah J., Ghillani P., Gunn C. (2021). IgA dominates the early neutralizing antibody response to SARS-CoV-2. Sci. Transl. Med..

[49] Fedele G., Russo G., Schiavoni I., Leone P., Olivetta E., Perri E., Zingaropoli M.A., Ciardi M.R., Pasculli P., Mastroianni C.M. (2022). Early IgG / IgA response in hospitalized COVID-19 patients is associated with a less severe disease. Diagn. Microbiol. Infect. Dis..

[50] Vossenkämper A., Blair P.A., Safinia N., Fraser L.D., Das L., Sanders T.J., Stagg A.J., Sanderson J.D., Taylor K., Chang F. (2013). A role for gut-associated lymphoid tissue in shaping the human B cell repertoire. J. Exp. Med..

[51] Victora G.D., Nussenzweig M.C. (2012). Germinal Centers. Ann. Rev. Immunol..

[52] Eisen H.N. (2014). Affinity enhancement of antibodies: How low-affinity antibodies produced early in immune responses are followed by high-affinity antibodies later and in memory B-cell responses. Cancer Immunol. Res..

[53] Correa V.A., Rodrigues T.S., Portilho A.I., Trzewikoswki de Lima G., De Gaspari E. (2021). Modified ELISA for antibody avidity evaluation: The need for standardization. Biomed. J..

[54] Bauer G. (2021). The potential significance of high avidity immunoglobulin G (IgG) for protective immunity towards SARS-CoV-2. Int. J. Infect. Dis..

[55] Iwasaki A., Yang Y. (2020). The potential danger of suboptimal antibody responses in COVID-19. Nat. Rev. Immunol..

[56] Klasse P.J., Sattentau Q.J. (2002). Occupancy and mechanism in antibody-mediated neutralization of animal viruses. J. Gen. Virol..

[57] Lu Y., Wang J., Li Q., Hu H., Lu J., Chen Z. (2021). Advances in Neutralization Assays for SARS-CoV-2. Scand. J. Immunol..

[58] Tan C.W., Chia W.N., Qin X., Liu P., Chen M.I.C., Tiu C., Hu Z., Chen V.C.W., Young B.E., Sia W.R. (2020). A SARS-CoV-2 surrogate virus neutralization test based on antibody-mediated blockage of ACE2–spike protein–protein interaction. Nat. Biotechnol..

[59] Yin Q., Zhang Y., Lian L., Qu Y., Wu W., Chen Z., Pei R., Chen T., Sun L., Li C. (2021). Chemiluminescence Immunoassay Based Serological Immunoassays for Detection of SARS-CoV-2 Neutralizing Antibodies in COVID-19 Convalescent Patients and Vaccinated Population. Viruses.

[60] Neumann F., Rose R., Römpke J., Grobe O., Lorentz T., Fickenscher H., Krumbholz A. (2021). Development of sars-cov-2 specific IgG and virus-neutralizing antibodies after infection with variants of concern or vaccination. Vaccines.

[61] von Rhein C., Scholz T., Henss L., Kronstein-Wiedemann R., Schwarz T., Rodionov R.N., Corman V.M., Tonn T., Schnierle B.S. (2021). Comparison of potency assays to assess SARS-CoV-2 neutralizing antibody capacity in COVID-19 convalescent plasma. J. Virol. Methods.

[62] Meyer B., Reimerink J., Torriani G., Brouwer F., Godeke G.J., Yerly S., Hoogerwerf M., Vuilleumier N., Kaiser L., Eckerle I. (2020). Validation and clinical evaluation of a SARS-CoV-2 surrogate virus neutralisation test (sVNT). Emerg. Microbes Infect..

[63] Cruvinel W.M., Júnior D.M., Araújo J.A.P., Catelan T.T.T., de Souza A.W.S., da Silva N.P., Andrade L.E.C. (2010). Immune system—Part I fundamentals of innate immunity with emphasis on molecular and cellular mechanisms of inflammatory response. Rev. Bras. Reumatol..

[64] Hogrefe W.R. (2005). Biomarkers and assessment of vaccine responses. Biomarkers.

[65] Freer G., Rindi L. (2013). Intracellular cytokine detection by fluorescence-activated flow cytometry: Basic principles and recent advances. Methods.

[66] Czerkinsky C.C., Svennerholm M.S. (1983). Ganglioside GM1 enzyme-linked immunospot assay for simple identification of heat-labile enterotoxin-producing *Escherichia coli*. J. Clin. Microbiol..

[67] Czerkinsky C.C., Nilsson L.A., Nygren H., Ouchterlony O., Tarkowski A. (1983). A solid-phase enzyme-linked immunospot (ELISPOT) assay for enumeration of specific antibody-secreting cells. J. Immunol. Methods.

[68] Janetzki S. (2016). ELISpot for Rookies (and Experts Too).

[69] Lima-Junior J., Morgado F., Conceição-Silva F. (2017). How Can Elispot Add Information to Improve Knowledge on Tropical Diseases?. Cells.

[70] Tripathy A.S., Vishwakarma S., Trimbake D., Gurav Y.K., Potdar V.A., Mokashi N.D., Patsute S.D., Kaushal H., Choudhary M.L., Tilekar B.N. (2021). Pro-inflammatory CXCL-10, TNF-α, IL-1β, and IL-6: Biomarkers of SARS-CoV-2 infection. Arch. Virol..

[71] Liu Y., Zhang C., Huang F., Yang Y., Wang F., Yuan J., Zhang Z., Qin Y., Li X., Zhao D. (2020). Elevated plasma levels of selective cytokines in COVID-19 patients reflect viral load and lung injury. Nat. Sci. Rev..

[72] Bösmüller H., Traxler S., Bitzer M., Häberle H., Raiser W., Nann D., Frauenfeld L., Vogelsberg A., Klingel K., Fend F. (2020). The evolution of pulmonary pathology in fatal COVID-19 disease: An autopsy study with clinical correlation. Virchows Arch..

[73] Hassaniazad M., Vahedi M.S., Samimagham H.R., Gharibzadeh A., Beyranvand S., Abassi H., Nikpoor A.R. (2021). Improvement of clinical outcome, laboratory findings and inflammatory cytokines levels using plasmapheresis therapy in severe COVID-19 cases. Respir. Med..

[74] Del Valle D.M., Kim-Schulze S., Huang H.H., Beckmann N.D., Nirenberg S., Wang B., Lavin Y., Swartz T.H., Madduri D., Stock A. (2020). An inflammatory cytokine signature predicts COVID-19 severity and survival. Nat. Med..

[75] Angioni R., Sánchez-Rodriguéz R., Munari F., Bertoldi N., Arcidiacono D., Cavinato S., Marturano D., Zaramella A., Realdon S., Cattelan A. (2020). Age-severity matched cytokine profiling reveals specific signatures in Covid-19 patients. Cell Death Res..

[76] Batista C.M., Foti L. (2021). Anti-SARS-CoV-2 and anti-cytokine storm neutralizing antibody therapies against COVID-19: Update, challenges, and perspectives. Int. Immunopharmacol..

[77] Mortaz E., Tabarsi P., Varahram M., Folkerts G., Adcock I.M. (2020). The Immune Response and Immunopathology of COVID-19. Front. Immunol..

[78] Li G., Fan Y., Lai Y., Han T., Li Z., Zhou P., Pan P., Wang W., Hu D., Liu X. (2020). Coronavirus infections and immune responses. J. Med. Virol..

[79] Foulds K.E., Wu C.Y., Seder R.A. (2006). Th1 memory: Implications for vaccine development. Immunol. Rev..

[80] Schwarzkopf S., Krawczyk A., Knop D., Klump H., Heinold A., Heinemann F.M., Thümmler L., Temme C., Breyer M., Witzke O. (2021). Cellular immunity in COVID-19 convalescents with PCR-confirmed infection but with undetectable SARS-CoV-2-specific IgG. Emerg. Infect. Dis..

[81] Li J., Hui A., Zhang X., Yang Y., Tang R., Ye H., Ji R., Lin M., Zhu Z., Türeci Ö. (2021). Safety and immunogenicity of the SARS-CoV-2 BNT162b1 mRNA vaccine in younger and older Chinese adults: A randomized, placebo-controlled, double-blind phase 1 study. Nat. Med..

[82] Folegatti P.M., Ewer K.J., Aley P.K., Angus B., Becker S., Belij-Rammerstorfer S., Bellamy D., Bibi S., Bittaye M., Clutterbuck E.A. (2020). Safety and immunogenicity of the ChAdOx1 nCoV-19 vaccine against SARS-CoV-2: A preliminary report of a phase 1/2, single-blind, randomised controlled trial. Lancet.

[83] Stephenson K.E., Le Gars M., Sadoff J., De Groot A.M., Heerwegh D., Truyers C., Atyeo C., Loos C., Chandrashekar A., McMahan K. (2021). Immunogenicity of the Ad26.COV2.S Vaccine for COVID-19. JAMA—J. Am. Med. Assoc..

[84] Simon D., Tascilar K., Schmidt K., Manger B., Weckwerth L., Sokolova M., Bucci L., Fagni F., Manger K., Schuch F. (2022). Brief Report: Humoral and cellular immune responses to SARS-CoV-2 infection and vaccination in B cell depleted autoimmune patients. Arthritis Rheumatol..

[85] Krammer F. (2021). A correlate of protection for SARS-CoV-2 vaccines is urgently needed. Nat. Med..

[86] Bauer G., Struck F., Schreiner P., Staschik E., Soutschek E., Motz M. (2021). The challenge of avidity determination in SARS-CoV-2 serology. J. Med. Virol..

[87] Struck F., Schreiner P., Staschik E., Wochinz-Richter K., Schulz S., Soutschek E., Motz M., Bauer G. (2021). Vaccination versus infection with SARS-CoV-2: Establishment of a high avidity IgG response versus incomplete avidity maturation. J. Med. Virol..

[88] Pratesi F., Caruso T., Testa D., Tarpanelli T., Gentili A., Gioè D., Migliorini P. (2021). Bnt162b2 mrna sars-cov-2 vaccine elicits high avidity and neutralizing antibodies in healthcare workers. Vaccines.

[89] Taylor S.C., Hurst B., Martiszus I., Hausman M.S., Sarwat S., Schapiro J.M., Rowell S., Lituev A. (2021). Semi-quantitative, high throughput analysis of SARS-CoV-2 neutralizing antibodies: Measuring the level and duration of immune response antibodies post infection/vaccination. Vaccine.

